# Overuse of tetanus toxoid vaccine: a common but under-addressed issue in Nepal

**DOI:** 10.1097/MS9.0000000000002525

**Published:** 2024-09-04

**Authors:** Suraj Shrestha, Roshan Aryal, Randhir S. Yadav, Sujita Baidya, Suman Acharya, Sanjeeb S. Bhandari

**Affiliations:** a Maharajgunj Medical Campus, Institute of Medicine, Kathmandu; b Kathmandu University School of Medical Sciences, Dhulikhel; c Mountain Medicine Society of Nepal, Kathmandu, Nepal

**Keywords:** adverse effects, booster, tetanus toxoid, tetanus, vaccine overuse

## Abstract

**Introduction and importance:**

Tetanus, though potentially fatal, is preventable with proper vaccination, but high tetanus titers from frequent or higher doses can lead to increased adverse events. In countries like Nepal, where tetanus vaccines are readily available over the counter, irrational and frequent dosing, especially in certain occupational groups, is a noted issue.

**Case presentation:**

A 28-year-old metal worker presented with a superficial cut on his forearm, managed with standard wound care, and reported a history of frequent tetanus vaccinations. Given his extensive vaccination history, a tetanus toxoid injection was deemed unnecessary, and he was educated on proper vaccination schedules and advised to seek medical attention for future injuries.

**Clinical discussion:**

Timely administration of vaccines for pre-exposure and postexposure prophylaxis is crucial for combating tetanus, with booster doses recommended every 10 years or as needed for wound management. High antibody titers from frequent tetanus vaccinations can increase the risk of adverse events, prompting guidelines to avoid administering Td more frequently than every 10 years unless necessary. Local reactions, like pain and swelling at the injection site, are common, while systemic reactions can include fever and peripheral neuropathy. Over-immunization is a concern in some regions, with frequent unnecessary booster doses potentially causing harm and highlighting the need for adherence to vaccination guidelines.

**Conclusion:**

Modifying and monitoring adult Td booster vaccination can lead to significant cost savings and fewer adverse events, requiring proper vaccination record-keeping, accurate assessment, and adherence to guidelines by healthcare workers.

## Introduction

Highlights

- Tetanus, though potentially fatal, is preventable with proper vaccination.
- In countries like Nepal, over-the-counter availability leads to irrational and frequent dosing, especially among certain occupational groups.
- High antibody titers from frequent vaccinations can cause adverse events, emphasizing the importance of adhering to the booster guideline.
- Proper monitoring and adherence to vaccination schedules can reduce costs and adverse events, highlighting the need for accurate vaccination record-keeping.
- Ensuring healthcare workers follow vaccination guidelines is crucial for preventing over-immunization and associated risks.

Tetanus is a serious and potentially fatal disease in unimmunized individuals without appropriate treatment^1,2^. Tetanus occurs by the introduction of spores of *Clostridium tetani* through contaminated wounds, lacerations, and abrasions^1^. Deep wounds with lacerated and bruised margins, devitalized tissue, and contaminated soil are at high risk of tetanus^1^. Toxins disseminate via blood and lymphatics which can interfere with the release of neurotransmitters and result in the symptoms^1^.

Approximately a million cases of tetanus are estimated to occur worldwide each year, with more than 200 000 deaths, mainly in low-income and middle-income countries^1^. Tetanus can only be prevented by vaccination because natural infections do not acquire immunity^1,2^. To sustain long-term protective immunity against tetanus, booster immunization is essential for adolescents and adults^2^. Even though there are numerous studies and reports on the immunization with tetanus vaccines, literature on overdose and too frequent vaccination with a booster is scarce. Here, we discuss the overuse of Tetanus Toxoid (TT) vaccine and the lack of adherence to guidelines with a brief example of biannual tetanus booster vaccine-seeking practice for the past several years. This case has been reported in line with Surgical CAse Report (SCARE) criteria^3^.

## Presentation of case

A 28-year-old metal worker male presented to the Emergency Department for wound care and TT vaccination after being cut with a sharp metal instrument. On examination, he had a superficial, clean cut on his right forearm with no active bleeding which was managed with standard wound care. On acquiring information on tetanus vaccination, he gave a history of being vaccinated with booster doses of TT every 6 months and additional doses after getting wounds while working with his machines and equipment for the past 4 years. He took the vaccines as over the counter with the last one being 3 months ago following a dog bite. However, there was no documentation of his vaccination status. He did not report any significant adverse events except for pain at the injection site during previous vaccinations. He reported about similar practices among his coworkers. His tetanus antibody titer was not tested due to the unavailability and unaffordability of the test. We deemed it not necessary to give him a TT injection, looking at his history of multiple vaccinations within the last 10 years, including recently. He was educated regarding the schedule and indication of the tetanus vaccine and was advised to seek medical attention after acquiring trauma.

## Discussion

Timely administration of vaccines for both pre-exposure, as well as postexposure prophylaxis, is instrumental to combat tetanus like any other vaccine-preventable disease^2^. The overall case-fatality rate among the vaccinated, the unvaccinated, and those with unknown vaccination history was estimated at 13.2%^2^. This indicates that tetanus is exceedingly rare, with a mortality rate of approximately three deaths per year among a population of >300 million^2^. Thus, there are clear guidelines for the administration of vaccines for tetanus prevention^2^.

In 1996, a revision of the tetanus/diphtheria vaccine schedule was adopted to address the vaccine-associated adverse effects when vaccines were administered at shorter intervals. Still, Hammarlund *et al*.^4^ have suggested revisiting this 10-year duration as the antibody titers are found to last longer. A monovalent vaccine is usually used only for booster or postexposure vaccination after an injury if there is a risk of tetanus infection^4,5^. After receipt of tetanus, diphtheria, and acellular pertussis (Tdap), booster doses of tetanus and diphtheria toxoids (Td) vaccine are recommended every 10 years or when indicated for wound management^5^. According to the CDC (Center for Disease Control and Prevention) and ACIP (Advisory Committee Immunization Practices), if a patient obtains a wound that is anything beyond ‘minor and uncontaminated’, they should receive another Td if it has been ≥5 years since their last vaccination^6^. Also, passive immunization using tetanus immune globulin (TIG), preferably of human origin, may be needed for prophylaxis in cases of dirty wounds in incompletely immunized patients^5^.

Melamed *et al*.^7^ did a clinical study on 16 soldiers who were mistakenly overdosed with tetanus vaccine found the mean tetanus antibody after 1 month of 153.75 +/- 82.5 IU/ml while it was 62.02 +/- 42.15 IU/ml in the control group. A titer greater than 0.01 IU/ml is considered protective^6^. Likewise, in 1995, 220 children in Eastern Europe were inadvertently given high-dose diphtheria and tetanus vaccine^8^. Among them, 94% were reported to have one or more vaccine-related reactions^8^.

Generally, the adverse effects following tetanus booster vaccination can be categorized as either local or systemic reactions^9^. Local reactions are the commonest and include pain at the site of injection, redness, and swelling of lymph nodes^9^. Systemic adverse events include myalgia, headache, fever, peripheral neuropathy, Guillain-Barre syndrome, anaphylaxis, etc.^9^. The preservative used in TT can lead to delayed-type hypersensitivity, while serum-sickness-like illnesses appear to be rarely associated with TT^9^. Jackson *et al*.^10^ found that medically attended local reactions were uncommon following Td vaccinations while the risk of such reactions was statistically significant in prior tetanus and diphtheria toxoid-containing vaccine recipients within 5 years.

Indeed, it has been shown that high prevaccination and postvaccination concentrations of tetanus and diphtheria antibodies increase the risk of adverse events after vaccination^9,10^. Local reactions, swelling of the lymph nodes, and fever, are more common among those with high tetanus antibody concentration^9^. In fact, it is estimated that tetanus antibody concentrations above 5 IU/ml would triple the risk of a reaction to vaccination^11,12^. These reactions are more severe with previous versions of the vaccines that were less purified and contained higher doses of toxoids^11^. In general, adverse reactions following TT may be influenced by the number of previous doses, the toxoid dosage, the route and method of administration, and the presence of adjuvants and/or other antigens in the preparation chosen^11–13^.

Administration of a large number of doses over a short time contributes to the severity of local reactions, possibly the result of high pre-existing antibody titers^14^. Such reactions are the basis for the general recommendation that Td is not given more frequently than every 10 years unless the patient requires tetanus prophylaxis for wound management as previously mentioned^6^. A study showed that the proportion of participants who reported local adverse events decreased from 86% for those with intervals of less than 5 years to 66% for those with intervals of 10 or more years^15^. In multivariate analyses, healthcare care workers who received the Tdap vaccine at an interval of less than 5 years after receipt of the Td vaccine were significantly more likely to experience local adverse events, compared with those who reported an interval of 10 or more years^15^. Females were found as an independent predictor of local adverse events after the Tdap vaccination^15^. Furthermore, it has revealed that an age of less than 30 years was significantly associated with higher rates of systemic adverse events^15^. Patients with a history of severe adverse reactions should not receive tetanus or diphtheria toxoid-containing vaccines more frequently than every 10 years. Extensive limb swelling affecting the entire limb where the vaccine was administered has also been reported^6^.

Even the administration of a booster dose to an individual who has completed five doses, even after 10 years is debatable because the excessive use of boosters could result in severe side effects^6,15^. Also, in Finland, after 50 years of decennial immunization, the reason for the increase in the number of adverse events may be linked to too high concentrations of prevaccination antibodies, especially for tetanus^16^. This questions whether current boosters are too frequent^16^. The United Kingdom does not recommend boosters to adults after the initial five-dose childhood immunization series. However, many countries, including the United States and Canada, continue booster vaccination every 10 years^17^.

Despite all these, the recommendation for an interval as short as 2 years is supported by a Canadian trial. Halperin *et al*.^18^ in an open-label, noninferiority clinical trial concluded that Tdap vaccination was well tolerated and may be safely given at intervals as short as 18 months after a previous tetanus and diphtheria toxoid-containing vaccine in children and adolescents. They found mild erythema and swellings in several cohorts that received Tdap in less than 10 years; however, more severe forms of reactions were noted noninferior^18^. It is unknown whether adults would demonstrate similar tolerability with shortened intervals; however, the previous CDC recommendations stated that safety could be inferred. In a mass vaccination campaign, intervals of less than 2 years between Td/TT and Tdap administration were found safe^19^. The available literature, although limited, does not identify major safety concerns with intervals of less than 2 years. There may be an increased incidence of injection site reactions and possibly fever with shortened intervals. Despite the concerns with the available evidence, the CDC is recommending vaccination with Tdap, regardless of the interval since the prior tetanus-containing vaccine, based on their assessment of risks and benefits^20^. Also, a booster dose of Tdap given ~10 years after a previous dose is well tolerated and immunogenic in adults aged 18 to <65 years^13^.

There is a paucity of data on the frequent use of tetanus vaccine booster doses. This can be a representative case of its nature found in different occupational groups. In Nepal, tetanus vaccines are easily available over the counter, and giving shots without acquiring detailed vaccination status is a quite common practice not only at pharmacies but also at many health facilities. Studies among clinicians have shown that the practice of giving Td to completely immunized patients with a clean wound who had received a booster less than 10 years previously is very common, accounting for as high as 70.8% of the over-immunization mistakes about tetanus immuno-prophylaxis^21^. In addition, physicians’ noncompliance with the guidelines for tetanus prophylaxis according to the past vaccination history and the kind of wound is another reason for over-immunization^22,23^. Illiteracy and unawareness of the rational use and adverse effects of vaccines further worsen the scenario. Nationwide availability of tetanus vaccines which are mostly imported is still a challenge. Furthermore, its overuse should be regarded as a serious concern from rational use and health hazard aspects as well as its unavailability for the needy ones.

## Conclusions

The advantage of modifying and monitoring adult Td booster vaccination includes not only substantial cost savings but also reduction of vaccine-associated adverse events due to hyperimmunization. This necessitates the proper record-keeping system of the vaccination status of the people of the country, eliciting history of previous vaccination, proper assessment of the wound site, and adherence to the provided guidelines by all the healthcare workers before booster administration.

## Ethical approval

Not applicable.

## Consent

Written informed consent was obtained from the patient for publication of this case report and accompanying images. A copy of the written consent is available for review by the Editor-in-Chief of this journal on request.

## Source of funding

Not applicable.

## Author contribution

S.S.: provided counseling and treatment to the patient, and contributed to collection of case information; S.S. and R.A.: conceptualization, collected all the required case information, reports; reviewed the literature and contributed in writing the original draft; S.S., R.A., R.S.Y., S.B., S.A., and S.S.B.: writing and editing the manuscript; S.A. and S.S.B.: senior author and manuscript reviewer. All the authors read and approved the final manuscript.

## Conflicts of interest disclosure

The authors declare no conflict of interest.

## Guarantor

Roshan Aryal.

## Data availability statement

Written informed consent was obtained from the patient for publication of this case report and accompanying images. A copy of the written consent is available for review by the Editor-in-Chief of this journal on request.

## Provenance and peer review

Not commissioned, externally peer-reviewed.
